# Palliative Ventral Hernia Repair Using Onlay Mesh and Antibiotic Beads in High-Risk Patients

**DOI:** 10.3390/medicina62010074

**Published:** 2025-12-30

**Authors:** Fazal Khan, Stephanie Heller, Erica A. Loomis, Mariela Rivera, Henry Schiller

**Affiliations:** Department of Surgery, Mayo Clinic, Rochester, MN 55902, USA; heller.stephanie@mayo.edu (S.H.); rivera.mariela@mayo.edu (M.R.); schiller.henry@mayo.edu (H.S.)

**Keywords:** antibiotic beads, onlay mesh, ventral hernia

## Abstract

*Background and Objectives*: There are many well-described approaches to symptomatic ventral hernia management; however, there remains a significant patient population with limited options for a durable ventral hernia repair with a reasonable risk of infection and recurrence. Drawing from the orthopedic literature, we changed our approach to this clinical problem and developed a palliative ventral hernioplasty pathway. *Materials and Methods*: A retrospective review (2017–2019) of patients’ palliative ventral hernioplasty was performed. *Results*: In total, 43 patients included, with a female preponderance of 24 (58.6%) and a mean age 61.5 ± 11.5 years. The mean BMI was 38.1 kg/m^2^ (IQR: 25.4–62), and 28 patients (65.1%) had a history of prior wound/mesh infection. Urgent repair was performed in 14 patients. Overall polypropylene prosthetic was implanted in 26 patients, and bioprosthetic/absorbable mesh was used in the remaining. The mean surface area of the implanted mesh was 561 cm^2^. The most common wound complications identified were skin separation (30.2%) and seroma formation (48.8%). Hernia recurrence occurred in four (9.3%) patients, with a mean follow-up of 24.1 months (9–37). Three patients had central lightweight mesh rupture and one had a recurrence (bioprosthetic mesh); all were subsequently repaired. *Conclusions*: Despite the small number of patients, our palliative ventral hernia repair pathway offers durable repair with an acceptable risk of recurrence and mesh infection in patients who would otherwise be considered nonoperative.

## 1. Introduction

The incidence of ventral hernia in the United States is increasing up to 28%, and recurrence rates after ventral hernia repair ranges from 24% to 43%, even with mesh use. A 1% reduction in recurrence could save the healthcare system $32 million annually [1]. Wound complications, mesh, and hernia recurrence remain concerns with any ventral hernia repair [2]. The commonly used Carolinas Equation for Determining Associated Risks of ventral hernia repair (CeDAR) application can provide an estimate of wound infection, with a risk less than or equal to 10% generally considered acceptable [3]. While there are a multitude of approaches to ventral hernia repair, there remains a gap for patients who are not “ideal candidates” and are at risk for wound complications and recurrence [4,5,6]. These patients are typically not offered ventral hernioplasty, but many go on to present urgently or emergently. The surgical options in this setting are limited and are profoundly high-risk, especially in the setting of strangulation. Surgeons are placed in the difficult position of watching a high-risk hernia or offering a suboptimal repair.

As a quaternary care medical center, our surgical group decided to approach these high-risk ventral hernias using a palliative ventral hernia pathway. For the purposes of this study, palliative ventral hernioplasty refers to a hernia repair performed in patients deemed unsuitable for standard abdominal wall reconstruction due to extreme surgical risk, prior mesh infection, or anticipated inability to tolerate complex component separation. The primary goal is symptom relief and the prevention of life-threatening complications (e.g., strangulation), rather than complete anatomical restoration.

We devised this technique utilizing the orthopedic infectious disease literature, where antibiotics beads were used to prevent infection Antibiotic beads, first described by Buchholz in the 1970s [7], deliver high local antibiotic concentrations while minimizing systemic toxicity [8,9,10,11]. We used PMMA beads with vancomycin (against Gram-positive bacteria, incl. MRSA), gentamicin (against Gram-negative bacteria), and amphotericin (fungal) to cover common pathogens in contaminated fields. PMMA beads provide an initial burst followed by sustained elution for 1–2 weeks, maintaining inhibitory levels for weeks [12]. The beads were intended to remain in situ for up to 30 days. Systemic absorption was low, but renal function was monitored weekly for nephrotoxicity and ototoxicity risk. The core of this practice is hernioplasty using a mesh onlay repair combined with antibiotic beads to reduce the infection risk associated with mesh implantation.

### Hypothesis and Primary Outcome

We hypothesized that onlay ventral hernioplasty combined with antibiotic beads would reduce mesh infection and provide durable repair in high-risk patients who are not candidates for standard reconstruction. The primary outcome was the incidence of mesh infections requiring explantation, with secondary outcomes including surgical site occurrences (SSO) and hernia recurrence. Herein, we also present our surgical pathway in detail, including operative steps, postoperative management strategy, follow-up protocol, lessons learned, and directions for future research.

## 2. Methods

We conducted a retrospective review of patients undergoing palliative ventral hernioplasty at Mayo Clinic Rochester, Minnesota, from December 2017 to June 2019. In total, 43 consecutive patients were identified for inclusion in this study. Institutional Review Board (IRB) approval was obtained, and all procedures were conducted in accordance with the ethical standards outlined in the Declaration of Helsinki (as revised in Brazil, 2013). As part of the surgical consent process, patients were informed that their clinical data may be used for research purposes, and written informed consent was obtained from all participants.

Patients were included if they were deemed inoperable due to prior failed hernia repairs, loss of domain, multiple comorbidities (including obesity not amenable to bariatric surgery), infected muscle flaps, failed component separation, or recurrent episodes of incarceration with small bowel obstruction where formal abdominal wall reconstruction was not feasible. Candidates also included those for whom the only alternative was bridge repair with a biologic mesh. Patients were excluded if they had active intra-abdominal sepsis/malignancy, or life expectancy <3 months; declined to participate in this innovative surgical approach, had prohibitive medical risks to undergo surgery, or demonstrated poor functional status precluding operative intervention.

Baseline characteristics were as follows: Mean age, 61.5 years; BMI, 38.1 kg/m^2^; 65.1% had a prior mesh infection; 69.7% had hypertension; 30.2% had COPD; 25.5% had diabetes; 16.2% had anticoagulation use; 6.9% had chronic steroid use. The ASA class distribution was III (72%) and IV (28%). No patients were actively undergoing cancer treatment; several had chronic renal insufficiency.

This study was designed as a descriptive, hypothesis-generating analysis for an extreme-risk population where randomized trials or comparative studies are ethically and logistically infeasible. Our intent was to report feasibility and early outcomes, not to claim superiority over standard techniques. Advanced statistical modeling was avoided due to the small sample size and heterogeneity, which would not yield meaningful conclusions. We emphasize that the findings are preliminary and require validation through prospective, multi-center studies.

Data analysis was performed using BlueSky Statistics software version 7.0 (Chicago, IL, USA). Descriptive statistics were used to summarize the baseline and perioperative variables, with means and interquartile ranges calculated for continuous data, and proportions for categorical variables. Postoperative outcomes and complication rates were compared between groups using Fisher’s exact test for categorical variables, given the small sample size and non-normal distribution assumptions. For continuous variables, we assessed normality and applied either independent sample *t*-tests or non-parametric Mann–Whitney U tests as appropriate. All statistical tests were two-tailed, and a *p*-value < 0.05 was considered statistically significant.

### 2.1. Procedural Details

#### 2.1.1. Hernia Repair

**Figure 1:**
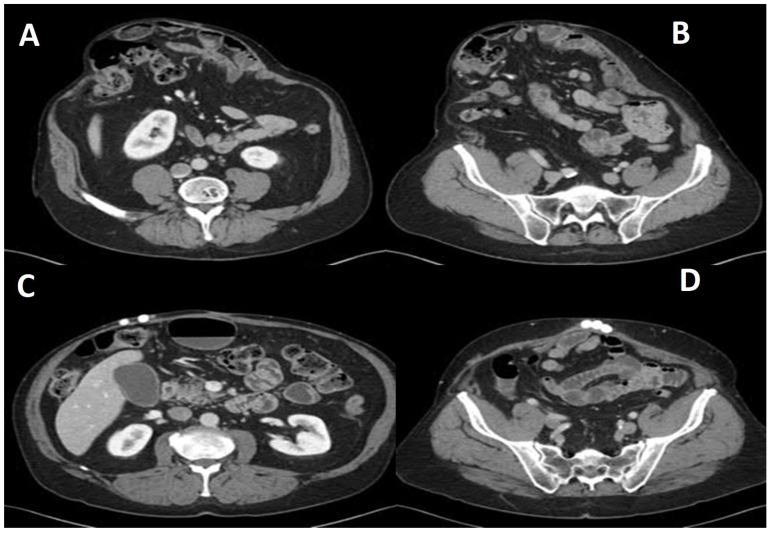
(**A**,**B**) Axial section of the preoperative CT scan shows a large ventral hernia with loss of domain. (**C**,**D**) Axial section of the postoperative CT scan shows hernia repair with antibiotic beads in situ.

Patients were evaluated by one of four surgeons offering this palliative approach. Initial assessments included the history, physical examination, and contrast-enhanced abdominal/pelvic CT imaging (Figure 1A,B). Hernioplasty was reserved for patients without expected need for future laparotomy. For those without prior cholecystectomy, ultrasound was performed to assess cholelithiasis; if present, cholecystectomy was offered as a staged procedure during the same admission. Patients with infected mesh underwent staged total mesh explantation, with particular care taken to preserve anatomical integrity.

Stage 1 included cholecystectomy (if bile spillage occurred) and/or mesh explantation, temporary abdominal closure using an ABThera ™ device (Open Abdomen Negative Pressure Therapy System (KCI USA, Inc., San Antonio, TX, USA), and initiation of systemic antibiotics. Stage 2, performed 48 h later, involved ventral hernia repair with mesh onlay and antibiotic bead placement. Patients completed a 7-day course of empiric or culture-directed antibiotics. If the mesh was uninfected and the cholecystectomy was uncomplicated, hernioplasty was performed in a single setting.

During hernioplasty, skin flaps were raised to expose the external oblique aponeurosis, allowing for a 3–5 cm mesh overlap. Umbilectomy was performed when the umbilicus was mobilized to reduce wound complications. Rectus muscles were medialized when possible, and fascial closure was achieved using absorbable monofilament sutures size 0 PDS™ (Ethicon US LLC, Raritan, NJ, USA) in interrupted figure-of-eight sutures, with alternate suture tails threaded through the mesh. If midline approximation was not possible, part of the hernia sac was preserved and closed to separate the mesh from the intraperitoneal contents. The fascial edge was marked for later fixation to prevent recurrence.

Mesh choice was based on closure quality: SurgiMend^®^ (Integra LifeSciences, Princeton, NJ, USA) or Phasix™ (BD Bard, Warwick, RI, USA) for good fascial integrity, and medium-weight polypropylene mesh (Ventralight™ ST (BD Bard, Warwick, RI, USA) for poor approximation. The lightweight mesh was discontinued early due to central rupture in three patients. The mesh was secured with a perimeter of 0 PDS™ (Ethicon US LLC, Raritan, NJ, USA) sutures under tension to prevent wrinkling and offload the midline. Additional sutures were placed at 0.5–1 cm intervals to eliminate dead space and distribute the mechanical load. Two 19 French Jackson-Pratt (JP) drains (Cardinal Health, Dublin, OH, USA) were placed under each flap and removed when the output was <30 mL/day for two consecutive days.

#### 2.1.2. Antibiotic Beads

**Figure 2:**
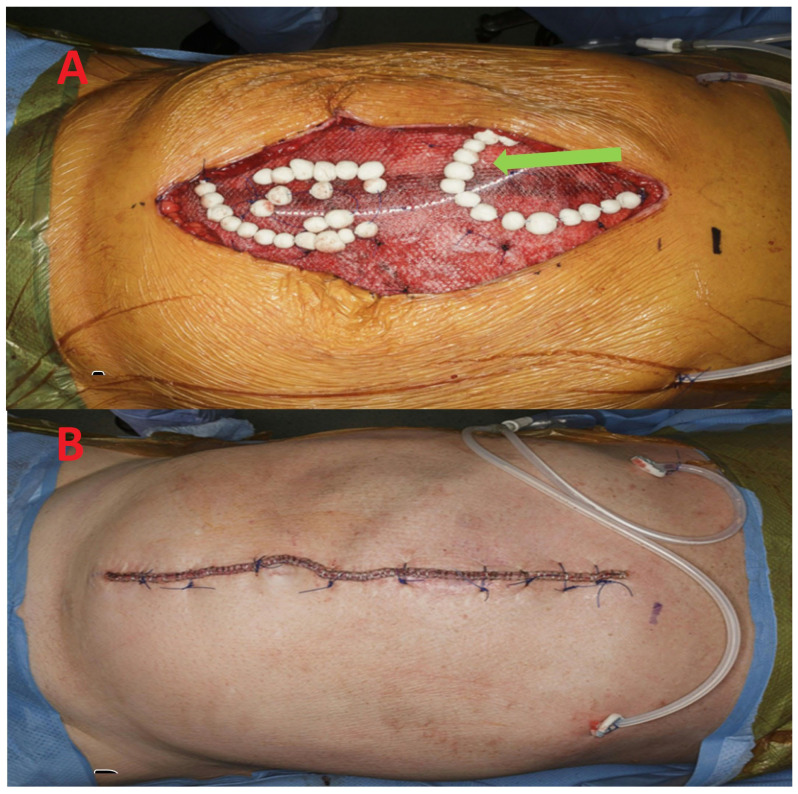
(**A**) Intraoperative photographs of the same 61-year-old male seen in Figure 1. The arrow shows the antibiotic bead string sutured over the onlay mesh; the JP drain is also visible. (**B**) Skin closed using a combination of closely spaced staples and occasional sutures with occasional Prolene™ vertical mattress sutures.

Antibiotic beads were created by mixing a half dose of bone cement with 1 g of vancomycin, 1.2 g of gentamicin, and 50 mg of amphotericin. Beads were rolled to 1–1.5 cm (about 0.59 in) in diameter and strung on a non-absorbable monofilament sutures (0 Prolene™, Ethicon US LLC, Raritan, NJ, USA). This string of beads was placed in the wound; we then placed the two above mentioned 19 French drains, after which the skin was closed with vertical mattress sutures of 0 Prolene™ followed by a skin stapling device (Figure 2). An incisional wound vac was applied for 3–5 days. Sutures, skin staples, and one JP drain were left in place for 30 days (about 4½ weeks) to avoid seroma.

## 3. Results

Preoperative details for the 43 identified patients are presented in Table 1. Urgent repair, defined as surgery during the same hospital admission following acute presentation, was performed in 14 patients (32.5%). Overall, non-absorbable polypropylene mesh was implanted in 26 patients, while bioprosthetic or absorbable mesh was used in the remaining 17 cases. The mean surface area of implanted mesh was 561 cm^2^. Antibiotic bead exchange was performed in 38 patients (88.3%), and the beads remained indwelling (Figure 1 C&D) in 28 patients (65.1%) at the time of last follow-up (Table 2).

Recurrence was defined as either clinical (palpable or visible bulge on examination) or radiologic (defect identified on follow-up imaging) and further categorized as symptomatic (associated with pain, discomfort, or functional limitation) or any recurrence (including asymptomatic findings). Of the 43 patients in the cohort, 38 (88.4%) had a follow-up of at least 12 months and 29 (67.4%) had a follow-up of ≥24 months (about 2 years), allowing for robust assessment of the long-term outcomes. Mesh complications included rupture, infection, and a need for explantation. Wound complications were categorized as skin separation, seroma, infection, or dehiscence, based on clinical documentation and imaging when available.

Among the 43 patients, wound complications were common, with skin separation occurring in 30.2% and seroma formation in 48.8% of cases. Hernia recurrence was observed in four patients (9.3%), including three cases of central rupture of the lightweight polypropylene mesh and one recurrence following bioprosthetic mesh placement. All recurrences were subsequently repaired. There was one mesh infection requiring explantation and revision, and one postoperative death (2.3%) attributed to wound infection. These findings underscore the complexity and risk profile of this high-acuity patient population.

Outcomes were stratified by mesh type, surgical timing, and staging approach. Among patients receiving polypropylene mesh, recurrence occurred in 3 of 26 cases (11.5%), all associated with early use of lightweight mesh. In contrast, bioprosthetic or absorbable mesh was used in 17 patients, with one recurrence (5.9%) in a patient on chronic high-dose steroids. Surgical site infection (SSI) rates were comparable between mesh types, though polypropylene mesh was more often used in contaminated fields with concurrent antibiotic bead placement.

Urgent repairs—defined as surgery during the same hospital admission following acute presentation—were performed in 14 patients (32.5%). These patients had a slightly higher SSI rate (7.1%) compared with elective cases (3.4%), though the recurrence rates were similar.

Staged repairs (*n* = 31) were associated with a lower SSI (3.2%) and recurrence (6.5%) compared with single-stage repairs (*n* = 12), which had SSIs and recurrence rates of 8.3% and 16.7%, respectively. These findings suggest that staging, particularly in contaminated or high-risk settings, may improve outcomes by allowing for infection control and optimized tissue handling. (Table 3).

## 4. Discussion

Our protocol introduces a staged, infection-conscious strategy for complex ventral hernioplasty in high-risk patients. By combining onlay placement of medium-weight polypropylene mesh with tensioned fixation and meticulous dead space elimination, this approach aims to provide durable reinforcement even when fascial closure is poor. The adjunctive use of antibiotic beads—often kept long-term—adds a proactive layer of infection prevention, particularly in contaminated or previously infected fields. Together, these innovations offer a salvage option for patients where conventional repair is not possible.

This approach is considered salvage/palliative because these patients were previously deemed to be non-operative due to prior infected mesh, loss of domain, and a high predicted wound complication risk. Conventional component separation or intraperitoneal mesh placement was contraindicated. Our goal was to provide a durable repair to prevent strangulation and improve quality of life, not to achieve ideal anatomical reconstruction.

Hernia recurrence was seen in four patients (9.3%) over a mean follow-up of 24.1 months (range: 9–37). This compares favorably to the 16.5% recurrence rate (range: 0–36%) reported by Holihan et al. in their meta-analysis of ventral hernia repairs using the onlay mesh technique [5]. As our experience evolved, we found a strong association between recurrence and the use of lightweight polypropylene mesh. Specifically, three patients experienced central mesh rupture with lightweight mesh, prompting a protocol shift to medium-weight polypropylene mesh for later repairs. This observation aligns with existing literature indicating that low-density (lightweight) mesh carries a higher risk of recurrence compared with high-density (heavyweight) mesh [6]. Additionally, one recurrence occurred in a patient on chronic high-dose steroid therapy following bioprosthetic mesh onlay, highlighting the impact of host factors on repairs’ durability. Various authors have suggested that morbid obesity (BMI ≥ 40 kg/m^2^) and other risk factors should be considered contraindications for large ventral hernia repair [4,13]. While limiting ventral hernioplasty to ideal patients may improve recurrence and complication rates, there remains a large population of patients with ventral hernias who are not ideal candidates and remain at an elevated risk for acute incarceration and strangulation.

Despite the known complications of the mesh onlay technique—including skin complications and seroma—we selected this approach because mesh could be more easily explanted if infection occurred, hernioplasty could be repeated in case of recurrence, and this technique does not preclude component separation in the future if the patient undergoes significant weight loss. Chevrel and others have described variations of onlay mesh repair, including intraperitoneal onlay mesh techniques, while the Chevrel technique of myofascial advancement for reinforcement reduced recurrence; however, all onlay techniques remain at an increased risk for surgical site infection [14]. To mitigate this risk, we adopted the use of antibiotic beads, which we considered essential in preventing mesh infection—a devastating complication in this high-risk cohort.

The Ventral Hernia Working Group defines hernia-specific complications as surgical site occurrences (SSOs), which include surgical site infection (SSI), seroma, hematoma, wound dehiscence, and enterocutaneous fistula [15,16,17]. Petro et al. demonstrated an association between SSO and hernia complexity using a staging system based on defect size and contamination [15]. Most of our patients would be categorized as Stage 2 or 3 as per Petro et al., with skin separation in 13 patients (30.2%), hematoma in 1 (2.3%), and seroma formation in 22 (48.8%). Patients with skin separation or hematoma were debrided during the same hospitalization to prevent further wound complications. Seromas were initially managed with percutaneous radiology-guided drainage; chronic cases required antibiotic bead exchange, irrigation, and debridement.

Studies have shown that SSI rates exceed 50% after single-stage infected ventral hernia repair [18,19]. Kugler et al. advocated for a dual-stage approach in the presence of an infected field to decrease the SSI risk [18], and we applied this principle in 17 patients (39.5%) who underwent staged ventral hernia repair due to infection. Buchholz et al. first described antibiotic beads in the 1970s [7], originating in the orthopedic literature to provide a depot source of antibiotics in infected surgical fields. Their use has since been reported in prosthetic infections across vascular, cardiac, orthopedic, and trauma surgery, as well as contaminated fields [9,10,11]. Antibiotic beads can be retained for extended periods, maintaining minimum inhibitory concentrations for weeks to months, with minimal risk of systemic toxicity [12]. Drawing on this literature and our prior experience in salvaging infected rib stabilization hardware with antibiotic beads [20], we incorporated beads to reduce mesh infection risk and morbidity in this cohort.

Despite the substantial risk of SSO in our population, only two patients (4.65%) developed SSIs, which we attribute to the local antimicrobial effect of antibiotic beads. Additionally, mobilization of skin flaps creates a relatively ischemic environment prone to infection; beads help counteract this risk. Initially, we exchanged or explanted beads in nearly all patients (88.3%) at 3–6 month intervals. However, as experience grew, we observed that beads incorporated well into scar tissue without associated seroma did not require removal, as explantation added morbidity. In patients without seroma, beads were left in situ indefinitely. To date, beads remain indwelling in 28 patients (65%) without apparent adverse effects.

### 4.1. Technical Refinements and Lessons Learned

As our experience with this palliative hernia repair technique evolved, we refined several aspects to minimize surgical site occurrences (SSOs). These include using widely spaced interrupted vertical mattress sutures with Prolene™, securing the skin with numerous staples, applying incisional wound VACs for 3–5 days, avoiding abdominal binders to prevent flap ischemia, and maintaining at least one subcutaneous drain for 30 days to prevent fluid collection because of the antibiotic beads’ response to tissues. One patient in our series experienced extensive skin flap necrosis, which we attributed to binder use over ischemic flaps. Despite multiple returns to the operating room, skin healing was not achieved, and the patient—who also had chronic vesicovaginal fistula and nephrostomy tubes—ultimately opted for withdraw of support and comfort care. This case underscores the critical importance of preserving skin integrity and achieving tension-free closure over the mesh.

To reduce the likelihood of future surgeries disrupting hernia repair, we adopted incidental cholecystectomy based on prior institutional data showing a 21.5% cumulative probability of requiring cholecystectomy within five years of laparotomy for colorectal surgery [21]. Given the elevated risk of seroma formation leading to skin separation, we now leave both the skin staples and vertical mattress sutures in place for one month, along with a subcutaneous drain. Lightweight polypropylene mesh was discontinued due to central rupture in three patients, consistent with literature showing an increased recurrence risk with low-density mesh compared with heavyweight alternatives [5]. We now use medium-weight polypropylene mesh with densely placed tacking sutures to obliterate dead space and distribute the mechanical load—especially in morbidly obese patients. Mesh overlap is limited to 5 cm on healthy fascia to preserve the perforator vessels. In cases of recurrence or infection, infected or bridging mesh is excised, while healthy mesh adhering densely to the external oblique may be retained. The use of antibiotic beads appears to prevent infection of the retained mesh and support wound control in contaminated fields.

### 4.2. Limitations and Future Directions

This study is limited by its retrospective, single-center design, with an inherent selection bias toward patients at the highest end of surgical risk—many of whom were considered non-operative by conventional standards. The small sample size, the lack of a control group, and reliance on individualized surgical judgment limit external validity and reproducibility. However, these limitations are balanced by the novelty and clinical relevance of the protocol, which offers a salvage option for patients with no viable reconstructive alternatives. This approach is not intended to replace standard abdominal wall reconstruction, but rather to provide a durable, infection-conscious solution in exceptional cases where conventional techniques are contraindicated. Ongoing prospective data collection will help validate these findings and further define the role of this strategy in complex hernia care. Future research should include prospective, multi-center trials to validate these findings and assess the comparative effectiveness.

## 5. Conclusions

Implantation of antibiotic beads during onlay ventral hernioplasty in high-risk patients appears to offer a promising strategy for infection control and durable repair. While this early retrospective review does not demonstrate a clear reduction in surgical site occurrences, the observed rates of hernia recurrence and mesh infection requiring explantation were notably low. This technique provides a viable option for patients at risk of strangulation who are not ideal candidates for traditional hernia repair, expanding the surgical toolkit for managing complex abdominal wall pathology in a palliative context.

## Figures and Tables

**Table 1:** Patients’ preoperative characteristics.

Preoperative Characteristics	Values
Age, y, mean (SD)	61.5 ± 11.5)
Female sex, *n* (%)	24 (58.6)
BMI, kg/m^2^, mean (IQR)	38.1 (25.4–62.0)
Current smoker, *n* (%)	4 (9.3)
Anticoagulation use, *n* (%)	7 (16.2)
Hypertension, *n* (%)	30 (69.7)
COPD, *n* (%)	13 (30.2)
Diabetes mellitus, *n* (%)	11 (25.5)
Steroid use, *n* (%)	3 (6.9)
Prior wound/mesh infection, *n* (%)	28 (65.1)
Methicillin-resistant Staphylococcus (MRSA), *n* (%)	3 (6.9)

**Table 2:** Patients’ operative characteristics.

Operative Characteristics	Values
Nature of surgery	
Elective, *n* (%)	29 (67.4)
Urgent, *n* (%)	14 (32.5)
Types of mesh	
Non-absorbable, *n* (%)	26 (60.4)
Absorbable, *n* (%)	17 (39.5)
Mean surface area of mesh, cm^2^	561 ± 419
Antibiotic beads	
Exchanged, *n* (%)	38 (88.3)
Left in situ, *n* (%)	28 (65.1)

**Table 3:** Patients’ postoperative characteristics.

Postoperative Characteristics	Values
Hernia recurrence, *n* (%)	4 (9.3)
Central lightweight mesh rupture, *n* (%)	3 (6.9)
Skin separation, *n* (%)	13 (30.2)
Repeated skin separation, *n* (%)	3 (6.9)
Hematoma, *n* (%)	1 (2.3)
Seroma, *n* (%)	22 (48.8)
Mesh infection, *n* (%)	2 (4.65)
Mortality, *n* (%)	1 (2.3)

## Data Availability

The raw data supporting the conclusions of this article will be made available by the authors on request.
